# Atezolizumab plus bevacizumab as first-line systemic therapy for hepatocellular carcinoma: a multi-institutional cohort study

**DOI:** 10.1093/oncolo/oyae142

**Published:** 2024-07-09

**Authors:** Michael H Storandt, Tyler J Zemla, Kanchi Patell, Nikolas Naleid, Jennifer J Gile, Nguyen H Tran, Sakti Chakrabarti, Zhaohui Jin, Mitesh Borad, Amit Mahipal

**Affiliations:** Department of Internal Medicine, Mayo Clinic, Rochester, MN, United States; Department of Quantitative Health Sciences, Mayo Clinic, Rochester, MN, United States; Department of Medical Oncology, University Hospitals Seidman Cancer Center and Case Western Reserve University, Cleveland, OH, United States; Department of Internal Medicine, University Hospitals Cleveland Medical Center, Cleveland, OH, United States; Department of Medical Oncology, Mayo Clinic, Rochester, MN, United States; Department of Medical Oncology, Mayo Clinic, Rochester, MN, United States; Department of Medical Oncology, University Hospitals Seidman Cancer Center and Case Western Reserve University, Cleveland, OH, United States; Department of Medical Oncology, Mayo Clinic, Rochester, MN, United States; Department of Medical Oncology, Mayo Clinic, Phoenix, AZ, United States; Department of Medical Oncology, University Hospitals Seidman Cancer Center and Case Western Reserve University, Cleveland, OH, United States

**Keywords:** hepatocellular carcinoma, atezolizumab, bevacizumab, Child-Pugh, ALBI

## Abstract

**Background:**

Atezolizumab plus bevacizumab is the standard of care for advanced hepatocellular carcinoma (HCC) in the first-line setting, although was only evaluated in patients with Child-Pugh (CP) A liver function in the IMbrave150 trial. We sought to determine the outcomes of these patients based on CP score and ALBI grade in the US population.

**Methods:**

This multicenter cohort study included patients with HCC who received atezolizumab with bevacizumab as first-line systemic therapy between March 2018 and November 2023. Overall survival (OS) was determined using the Kaplan-Meier method and multivariate analyses were performed using Cox proportional hazard regression method.

**Results:**

Among 322 patients, 226, 86, and 10 patients had CP-A, CP-B, and CP-C liver function, respectively. Median age was 66.5 years, 78.6% were male, and 82.6% were White. Median OS (mOS) was 21.6 months for those with CP-A, 9.1 months for those with CP-B7, and 4.7 months for those with CP-B8-C12 (*P* < .0001). Among patients with CP-A, those with ALBI grade 1 had an mOS of 34.9 months versus 14.2 months in those with grade 2. In multivariate analyses, CP score, ALBI grade, hepatitis B, performance status, and macrovascular invasion were significantly associated with survival.

**Conclusions:**

CP score is an important prognostic tool for patients with HCC receiving atezolizumab plus bevacizumab, and this regimen remains a viable option for patients with CP-B7 with no additional safety concern, although the benefit is significantly less than those with CP-A. ALBI score has independent predictive value in patients with CP-A liver function.

## Teaser

This multi-institution retrospective cohort study focused on patients with advanced hepatocellular carcinoma who received atezolizumab plus bevacizumab in the first-line setting, evaluating patient outcomes based on multiple patient and tumor factors, with a focus on baseline liver function.

**Implications for Practice**

This multi-institutional cohort study evaluated the outcomes of patients with hepatocellular cancer who received atezolizumab plus bevacizumab as first-line systemic therapy. This study included patients who would have been ineligible for IMBrave150 trial. Patients with Child-Pugh (CP) B7 score derived benefit from treatment without additional safety concerns, although survival was less than those with CP-A. Patients with CP score ≥ 8 should not receive systemic therapy. ALBI score was predictive of outcomes in patients with CP-A and should be routinely used in clinical practice. Black patients seem to have poorer outcomes and future trials should focus on minority population.

## Introduction

Approximately 90% of patients diagnosed with hepatocellular carcinoma (HCC) have some form of chronic liver disease.^1^ The Child-Turcotte score was first developed in 1964 as a means to predict mortality of patients undergoing portocaval shunt surgery, and was computed using subjective factors including encephalopathy, nutritional status, and ascites, as well as objective factors including serum albumin and bilirubin.^2^ This score was later modified by Pugh to include the international normalized ratio (INR) in place of nutritional status, hence why the score is now commonly referred to as the Child-Pugh (CP) score.^3,4^ CP score has been incorporated in the Barcelona Clinic Liver Cancer staging system, which guides therapeutic options for patients with HCC based on tumor spread and liver function.^5^

Systemic therapy for advanced HCC has been primarily evaluated in patients with CP class A liver function due to concurrent mortality risk in patients with higher liver dysfunction. Currently, the combination of atezolizumab and bevacizumab is a standard of care option in the first-line setting for eligible patients with advanced HCC based on the IMbrave150 clinical trial, which demonstrated survival benefit compared to sorafenib.^6^ However, this trial was limited to patients with CP class A liver function, with 72% having CP-A5 and 28% having CP-A6 liver function. At this time, there is little evidence to support the use of this combination in patients with CP-B or CP-C liver function. Moreover, there is limited data regarding outcomes of treatment of HCC with atezolizumab plus bevacizumab in Western populations. In the IMbrave150 trial, 57% of patients were Asian and 35% of patients were White. Further, only 10 Black/African American patients were enrolled in this trial, limiting the generalizability of results in this patient population. Other FDA-approved first-line systemic therapeutic options include tremelimumab plus durvalumab, lenvatinib, and sorafenib, but these are less frequently used compared to atezolizumab plus bevacizumab.

More recently, the ALBI score has gained favor as a simplified model for liver function analysis. This score is calculated using serum albumin and bilirubin levels, and excludes subjective measures that are required to calculate a CP score.^7^ ALBI score has been demonstrated to have prognostic value in patients with cirrhosis. ALBI has been shown to have a good correlation with CP and the model of the end-stage liver disease (MELD) scores.^8^ The predictive value of ALBI in assessing patient outcomes when undergoing treatment with atezolizumab plus bevacizumab for HCC is not well known at this time.

In this study, we conducted a multi-institution retrospective cohort study of patients with advanced HCC who received atezolizumab plus bevacizumab in the first-line setting in the United States and evaluated patients’ outcomes based on multiple patient and tumor factors, with a focus on baseline liver function.

## Methods

### Study design and patient selection

We conducted a multisite, multi-institution retrospective analysis of patients seen within the Mayo Clinic Health system (with sites in Minnesota, Wisconsin, Iowa, Florida, and Arizona) and University Hospitals Siedman Cancer Center and Case Western Reserve University (with sites in Ohio). Patients were included who were ≥18 years of age, had a prior diagnosis of HCC and planned to receive atezolizumab plus bevacizumab as first-line systemic therapy from March 1, 2018, to November 1, 2023. Pathologic confirmation of HCC was not required if the diagnosis was based on the Liver Imaging Reporting and Data System.^9^ The keywords used to retrieve the data were International Classification of Diseases-10-CM code of “C20.0” and/or “hepatocellular cancer.” Patients were excluded if they had mixed hepatocellular cancer-cholangiocarcinoma or had more than one primary malignancy requiring concurrent treatment. Exclusion criteria included patients who had received systemic therapy for HCC prior to receiving atezolizumab plus bevacizumab. All cases were reviewed at multidisciplinary tumor boards at respective sites and were considered having advanced disease not amenable to locoregional liver-directed therapy.

Eastern Cooperative Oncology Group (ECOG) performance score, laboratory data at the time of diagnosis including albumin, bilirubin, and INR, prior treatment history, and patient demographics were collected by retrospective review of electronic medical records. Response was assessed using Response Evaluation Criteria in Solid Tumors v 1.1. Response assessment was performed by 2 authors (M.H.S. and A.M.). This research was conducted ethically in accordance with the World Medical Association Declaration of Helsinki. The study protocol was reviewed and approved by Case Western Reserve University and Mayo Clinic Institutional Review Board.

### Analysis

Patient demographics were compared across CP score levels. For categorical variables, frequencies (percentages) were calculated overall and within CP score levels. For continuous variables, mean and standard deviation (SD) were calculated overall and within CP score levels. Analysis of both overall survival (OS) and progression-free survival (PFS) was performed using the Kaplan-Meier method. OS was defined as the time from the date of first treatment with atezolizumab plus bevacizumab till death. If patients were alive at the last follow-up, they were censored on that date. PFS was defined as the time from the date of treatment to the time of progression or death from any cause. If a patient was alive and progression-free, they were censored at the date of last follow-up. Both OS and PFS were compared across CP liver status using the log-rank *P*-value. Additionally, a subgroup analysis of Child-Pugh A patients was performed to compare both OS and PFS based on ALBI scores. A univariate analysis was performed for both PFS and OS to identify prognostic factors. Factors to be included in the univariable analysis were determined a priori and the covariates that had a significance level of <0.2 were included in the candidate factors to be used in the multivariable analysis. A backward selection model (using *P*-value threshold for remaining in a model of .05) was performed to determine the final multivariable model for both OS and PFS. All statistical analysis was performed using SAS v9.4.

## Results

### Cohort characteristics

We identified 322 patients with HCC who planned to receive atezolizumab plus bevacizumab as first-line systemic therapy, with calculable CP liver function. Fourteen patients were excluded from efficacy analysis due to the inability to calculate CP score based on available data, and 5 patients were excluded due to a synchronous, non-HCC malignancy. In our cohort, 22 (6.8%) patients received atezolizumab alone without bevacizumab primarily due to large varices that could not be treated in time to initiate systemic therapy. Median age was 66.5 years, 78.6% were male, and 82.6% were White. The study population included 28 (8.7%) Black/African American patients. Risk factors for HCC included hepatitis B (5.3%), hepatitis C (34.8%), alcohol use (26.1%), and metabolic-associated steatohepatitis (MASH, 15.5%). At time of starting treatment, 70.2% had CP-A, 26.7% had CP-B, and 3.1% had CP-C liver function, while 37.3% had ALBI grade 1, 55.0% had ALBI grade 2, and 7.8% had ALBI grade 3 liver function. CP score was reported in a patient's chart by a treating provider in 68.3% of patients and was concordant with the retrospectively calculated CP score used in this analysis in 80.9% of the patients. Prior treatment included radiation therapy (11.2%), embolization (42.9%), ablation (17.4%), and surgery (14.6%). Extrahepatic metastases were present in 30.4% of the patients. Baseline patient and tumor characteristics are described in Table 1.

**Table 1.** Patient characteristics, prior local therapies, and baseline liver function.

	CP-A (*N* = 226)	CP-B (*N* = 86)	CP-C (*N* = 10)	Total (*N* = 322)
Age at diagnosis (years), median (IQR)	67.0 (61.0, 73.0)	66.0 (61.0, 72.0)	62.5 (59.0, 67.0)	66.5 (61.0, 72.0)
<65	86 (38.1%)	37 (43.0%)	6 (60.0%)	129 (40.1%)
≥65, <75	94 (41.6%)	35 (40.7%)	4 (40.0%)	133 (41.3%)
≥75	46 (20.4%)	14 (16.3%)	0 (0.0%)	60 (18.6%)
Sex				
Female	54 (23.9%)	14 (16.3%)	1 (10.0%)	69 (21.4%)
Male	172 (76.1%)	72 (83.7%)	9 (90.0%)	253 (78.6%)
Race				
White	188 (83.2%)	69 (80.2%)	9 (90.0%)	266 (82.6%)
African American	19 (8.4%)	9 (10.5%)	0 (0.0%)	28 (8.7%)
Asian	10 (4.4%)	3 (3.5%)	1 (10.0%)	14 (4.3%)
Unknown/other	8 (3.5%)	4 (4.7%)	0 (0.0%)	12 (3.7%)
American Indian	1 (0.4%)	1 (1.2%)	0 (0.0%)	2 (0.6%)
BMI, kg/m^2^				
<25	64 (28.3%)	25 (29.1%)	3 (30.0%)	92 (28.6%)
≥25, <30	69 (30.5%)	35 (40.7%)	3 (30.0%)	107 (33.2%)
≥30	93 (41.2%)	26 (30.2%)	4 (40.0%)	123 (38.2%)
Hepatitis B				
No	216 (95.6%)	80 (93.0%)	9 (90.0%)	305 (94.7%)
Yes	10 (4.4%)	6 (7.0%)	1 (10.0%)	17 (5.3%)
Hepatitis C				
No	149 (65.9%)	53 (61.6%)	8 (80.0%)	210 (65.2%)
Yes	77 (34.1%)	33 (38.4%)	2 (20.0%)	112 (34.8%)
Alcohol use				
No	173 (76.5%)	60 (69.8%)	5 (50.0%)	238 (73.9%)
Yes	53 (23.5%)	26 (30.2%)	5 (50.0%)	84 (26.1%)
MASH				
No	195 (86.3%)	68 (79.1%)	9 (90.0%)	272 (84.5%)
Yes	31 (13.7%)	18 (20.9%)	1 (10.0%)	50 (15.5%)
Other risk factors for cirrhosis				
No	215 (95.1%)	84 (97.7%)	9 (90.0%)	308 (95.7%)
Yes	11 (4.9%)	2 (2.3%)	1 (10.0%)	14 (4.3%)
ECOG PS				
0	102 (45.1%)	20 (23.3%)	2 (20.0%)	124 (38.5%)
1	111 (49.1%)	52 (60.5%)	5 (50.0%)	168 (52.2%)
2	13 (5.8%)	13 (15.1%)	3 (30.0%)	29 (9.0%)
3	0 (0.0%)	1 (1.2%)	0 (0.0%)	1 (0.3%)
Macrovascular invasion				
No	139 (61.5%)	41 (47.7%)	4 (40.0%)	184 (57.1%)
Yes	87 (38.5%)	45 (52.3%)	6 (60.0%)	138 (42.9%)
Extrahepatic metastasis				
No	154 (68.1%)	64 (74.4%)	6 (60.0%)	224 (69.6%)
Yes	72 (31.9%)	22 (25.6%)	4 (40.0%)	98 (30.4%)
AFP, ng/mL				
Median (IQR)	29.5 (5.4, 932.5)	158 (14.0-1411.0)	385.5 (11.0, 3462.0)	51.0 (7.9, 1147.0)
Prior embolization				
No	119 (52.7%)	57 (66.3%)	8 (80.0%)	184 (57.1%)
Yes	107 (47.3%)	29 (33.7%)	2 (20.0%)	138 (42.9%)
Prior ablation				
No	179 (79.2%)	77 (89.5%)	10 (100.0%)	266 (82.6%)
Yes	47 (20.8%)	9 (10.5%)	0 (0.0%)	56 (17.4%)
Prior radiotherapy				
No	200 (88.5%)	76 (88.4%)	10 (100.0%)	286 (88.8%)
Yes	26 (11.5%)	10 (11.6%)	0 (0.0%)	36 (11.2%)
Prior surgery				
No	183 (81.0%)	83 (96.5%)	9 (90.0%)	275 (85.4%)
Yes	43 (19.0%)	3 (3.5%)	1 (10.0%)	47 (14.6%)
First-line therapy				
Atezo + Bev	214 (94.7%)	79 (91.9%)	7 (70.0%)	300 (93.2%)
Atezo alone	12 (5.3%)	7 (8.1%)	3 (30.0%)	22 (6.8%)
Age at start of treatment, mean (SD)	67.2 (10.4)	67.2 (8.4)	60.8 (9.6)	67.0 (9.9)
<65	74 (32.7%)	34 (39.5%)	6 (60.0%)	114 (35.4%)
≥65, <75	99 (43.8%)	34 (39.5%)	4 (40.0%)	137 (42.5%)
≥75	53 (23.5%)	18 (20.9%)	0 (0.0%)	71 (22.0%)
Albumin (g/dL)				
<2.8	0 (0.0%)	12 (14.0%)	6 (60.0%)	18 (5.6%)
2.8-3.5	41 (18.1%)	59 (68.6%)	3 (30.0%)	103 (32.0%)
>3.5	185 (81.9%)	15 (17.4%)	1 (10.0%)	201 (62.4%)
Bilirubin (mg/dL)				
<2	218 (96.5%)	53 (61.6%)	0 (0.0%)	271 (84.2%)
2-3	8 (3.5%)	24 (27.9%)	3 (30.0%)	35 (10.9%)
>3	0 (0.0%)	9 (10.5%)	7 (70.0%)	16 (5.0%)
Ascites				
Absent	183 (81.0%)	22 (25.6%)	1 (10.0%)	206 (64.0%)
Slight	43 (19.0%)	44 (51.2%)	5 (50.0%)	92 (28.6%)
Moderate	0 (0.0%)	20 (23.3%)	4 (40.0%)	24 (7.5%)
Encephalopathy				
None	226 (100.0%)	83 (96.5%)	10 (100.0%)	319 (99.1%)
Grade 1-2	0 (0.0%)	3 (3.5%)	0 (0.0%)	3 (0.9%)
INR				
<1.7	225 (99.6%)	84 (97.7%)	6 (60.0%)	315 (97.8%)
1.7-2.3	1 (0.4%)	1 (1.2%)	4 (40.0%)	6 (1.9%)
>2.3	0 (0.0%)	1 (1.2%)	0 (0.0%)	1 (0.3%)
ALBI score, mean (SD)	−2.6 (0.4)	−1.8 (0.4)	−1.1 (0.4)	−2.3 (0.6)
ALBI grade				
1	117 (51.8%)	3 (3.5%)	0 (0.0%)	120 (37.3%)
2	109 (48.2%)	65 (75.6%)	3 (30.0%)	177 (55.0%)
3	0 (0.0%)	18 (20.9%)	7 (70.0%)	25 (7.8%)
CP score				
5	135 (59.7%)	0 (0.0%)	0 (0.0%)	135 (41.9%)
6	91 (40.3%)	0 (0.0%)	0 (0.0%)	91 (28.3%)
7	0 (0.0%)	49 (57.0%)	0 (0.0%)	49 (15.2%)
8	0 (0.0%)	25 (29.1%)	0 (0.0%)	25 (7.8%)
9	0 (0.0%)	12 (14.0%)	0 (0.0%)	12 (3.7%)
10	0 (0.0%)	0 (0.0%)	9 (90.0%)	9 (2.8%)
12	0 (0.0%)	0 (0.0%)	1 (10.0%)	1 (0.3%)

Abbreviations: CP, Child-Pugh; BMI, body mass index; MASH, metabolic-associated steatohepatitis; PS, performance status; AFP, alpha fetoprotein.

### Overall survival by baseline liver function

**Figure 1.**
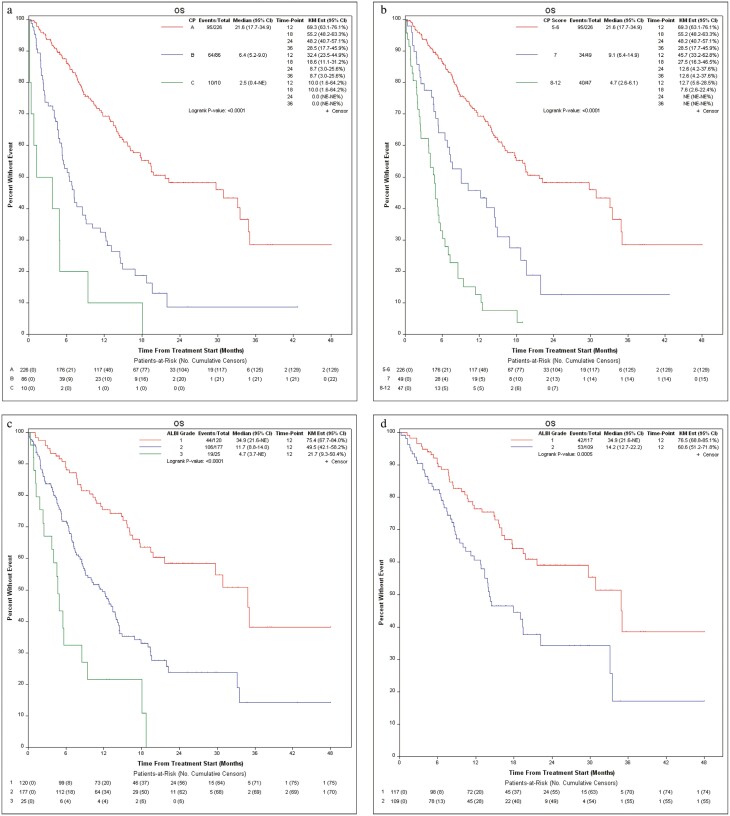
Overall survival by (a) Child-Pugh liver status (A vs B vs C), (b) Child-Pugh liver status (A5/6 vs B7 vs B8-C12), (c) ALBI liver function, and (d) in patients with Child-Pugh A liver function by ALBI grade.

Patients with CP-A liver function manifested a median OS (mOS) of 21.6 months (95% CI, 17.7-34.9), followed by patients with CP-B liver function at 6.4 months (95% CI, 5.2-9.0) and CP-C liver function at 2.5 months (95% CI, 0.4-NE; *P* < .0001; Figure 1a). Further stratifying patients with CP-B liver function, those with CP-B7 had an mOS of 9.1 months (95% CI, 6.4-14.9), CP-B8 had an mOS of 5.5 months (95% CI, 3.8-11.4), and CP-B9 had an mOS of 4.0 months (95% CI, 1.8-NE). Collectively, those with CP-B8-C12 liver function had an mOS of 4.7 months (95% CI, 2.6-6.1; Figure 1b). OS at 12, 18, 24, and 36 months was 69.3%, 55.2%, 48.2%, and 28.5% for those with CP-A; 45.7%, 27.5%, 12.6%, and 12.6% for those with CP-B7; and 12.7%, 7.6%, not evaluable (NE), and NE for those with CP-B8-C12.

Next, evaluating survival using ALBI score, patients with ALBI grade 1 liver function had an mOS of 34.9 months (95% CI, 21.6-NE), those with grade 2 had an mOS of 11.7 months (95% CI, 8.8-14.0), and those with grade 3 had an mOS of 4.7 months (95% CI, 3.7-NE; *P* < .0001; Figure 1c). When looking only at a subset of patients with CP-A liver function, those with ALBI grade 1 had an mOS of 34.9 months (95% CI, 21.6-NE) while those with ALBI grade 2 had an mOS of 14.2 months (95% CI, 12.7-22.2; *P* = .0005; Figure 1d).

### Progression-free survival by baseline liver function

**Figure 2.**
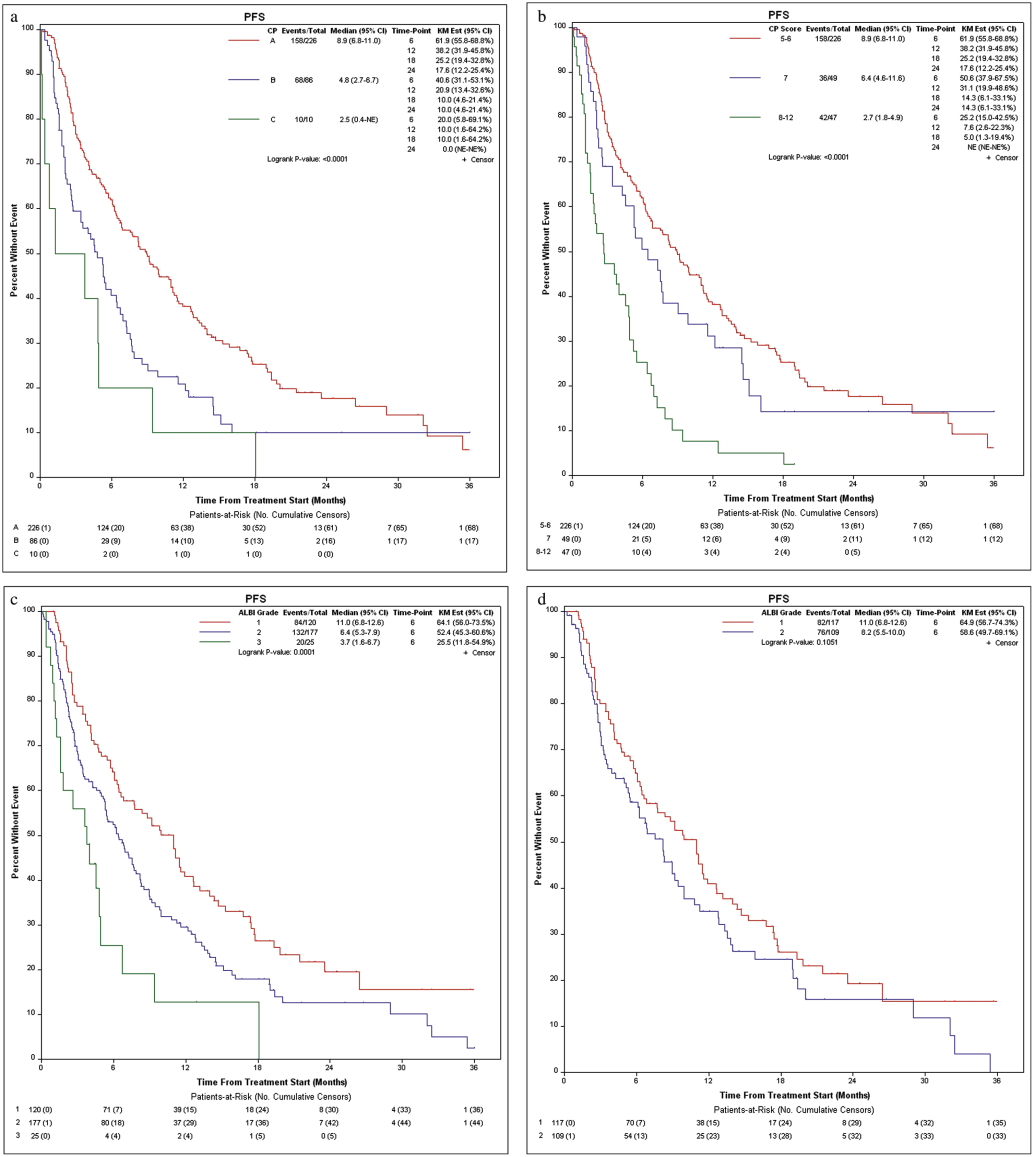
Progression-free survival by (a) Child-Pugh liver status (A vs B vs C), (b) Child-Pugh liver status (A5/6 vs B7 vs B8-C12), (c) ALBI liver function, and (d) in patients with Child-Pugh A liver function by ALBI grade.

Next, we evaluated PFS based on baseline liver function. Patients with CP-A liver function had an mPFS of 8.9 months (95% CI, 6.8-11.0), those with CP-B had an mPFS of 4.8 months (95% CI, 2.7-6.7), and those with CP-C had an mPFS of 2.5 months (95% CI, 0.4-NE; *P* < .0001; Figure 2a). When further subdividing patients with CP-B, those with CP-B7 had an mPFS of 6.4 months (95% CI, 4.6-11.6), CP-B8 had an mPFS of 2.7 months (95% CI, 1.8-7.2), and CP-B9 had an mPFS of 2.7 months (95% CI, 1.1-NE). Among patients with CP-B8-C12, mPFS was 2.7 months (95% CI, 1.8-4.9; Figure 2b). PFS at 6, 12, 18, and 24 months was 61.9%, 38.2%, 25.2%, and 17.6% in those with CP-A; 50.6%, 31.1%, 14.3%, and 14.3% in those with CP-B7; and 25.2%, 7.6%, 5.0%, and NE in those with CP-B8-C12.

When evaluating PFS by ALBI grade, mPFS was 11.0 months (95% CI, 6.8-12.6), 6.4 months (95% CI, 5.3-7.9), and 3.7 months (95% CI, 1.6-6.7) for grades 1, 2, and 3, respectively (*P* = .0001; Figure 2c). When looking only at patients with CP-A liver function, those with ALBI grade 1 had an mPFS of 11.0 months (95% CI 6.8-12.6) while those with grade 2 had an mPFS of 8.2 months (95% CI 5.5-10.0; *P* = .1051; Figure 2d).

### Response and adverse events by baseline liver function

A complete response (CR) was seen in 3.4% of patients, including 4.4% with CP-A and 1.2% with CP-B liver function. A partial response (PR) was seen in 29.2%, 19.8%, and 10.0% with CP-A, CP-B, and CP-C, respectively, with stable disease (SD) in 32.7%, 33.7%, and 40.0%, respectively. A disease control rate of 66.4%, 54.7%, and 50% was seen in CP-A, CP-B, and CP-C, respectively. Responses are listed in Table 2.

**Table 2.** Response rate, adverse events, and second-line therapy by baseline liver function.

	CP-A (*N* = 226)	CP-B (*N* = 86)	CP-C (*N* = 10)	Total (*N* = 322)
Best response				
CR	10 (4.4%)	1 (1.2%)	0 (0.0%)	11 (3.4%)
PR	66 (29.2%)	17 (19.8%)	1 (10.0%)	84 (26.1%)
SD	74 (32.7%)	29 (33.7%)	4 (40.0%)	107 (33.2%)
PD	64 (28.3%)	37 (43.0%)	5 (50.0%)	106 (32.9%)
NE	12 (5.3%)	2 (2.3%)	0 (0.0%)	14 (4.3%)
Hypertension (grade)				
None	175 (77.4%)	74 (86.0%)	10 (100.0%)	259 (80.4%)
1	28 (12.4%)	9 (10.5%)	0 (0.0%)	37 (11.5%)
2	12 (5.3%)	2 (2.3%)	0 (0.0%)	14 (4.3%)
3	10 (4.4%)	1 (1.2%)	0 (0.0%)	11 (3.4%)
4	1 (0.4%)	0 (0.0%)	0 (0.0%)	1 (0.3%)
Bleeding				
No	203 (89.8%)	72 (83.7%)	8 (80.0%)	283 (87.9%)
Yes	23 (10.2%)	14 (16.3%)	2 (20.0%)	39 (12.1%)
Fatigue				
No	88 (38.9%)	33 (38.4%)	8 (80.0%)	129 (40.1%)
Yes	138 (61.1%)	53 (61.6%)	2 (20.0%)	193 (59.9%)
Immune-related AE				
No	192 (85.0%)	70 (81.4%)	8 (80.0%)	270 (83.9%)
Yes	34 (15.0%)	16 (18.6%)	2 (20.0%)	52 (16.1%)
Max grade of immune-related AE				
0	192 (85.0%)	71 (82.6%)	8 (80.0%)	271 (84.2%)
1	11 (4.9%)	2 (2.3%)	1 (10.0%)	14 (4.3%)
2	13 (5.8%)	8 (9.3%)	1 (10.0%)	22 (6.8%)
3	7 (3.1%)	5 (5.8%)	0 (0.0%)	12 (3.7%)
4	1 (0.4%)	0 (0.0%)	0 (0.0%)	1 (0.3%)
5	2 (0.9%)	0 (0.0%)	0 (0.0%)	2 (0.6%)
Second-line therapy				
Yes				
Lenvatinib	57 (25.4%)	16 (18.6%)	0 (0.0%)	73 (22.8%)
Cabozantinib	7 (3.1%)	4 (4.7%)	0 (0.0%)	11 (3.4%)
Sorafenib	5 (2.2%)	1 (1.2%)	0 (0.0%)	6 (1.9%)
Regorafenib	2 (0.9%)	0 (0.0%)	0 (0.0%)	2 (0.6%)
Tremelimumab + Durvalumab	5 (2.2%)	0 (0.0%)	0 (0.0%)	5 (1.6%)
Ipilimumab + Nivolumab	5 (2.2%)	3 (3.5%)	0 (0.0%)	8 (2.5%)
Durvalumab	0 (0.0%)	1 (1.2%)	0 (0.0%)	1 (0.3%)
Others	12 (5.4%)	1 (1.2%)	0 (0.0%)	13 (4.1%)
No	131 (58.5%)	60 (69.8%)	10 (100.0%)	201 (62.8%)
Missing	2	0	0	2

Abbreviations: CR, complete response; PR, partial response; SD, stable disease; PD, progressive disease; NE, not evaluable; AE, adverse event.

Among patients with a CR/PR, mOS was not reached (95% CI, 29.1-NE), with a 12-month OS of 86.5%. PFS in this group was 16.1 months (95% CI, 14.0-26.4). Those with SD manifested an mOS of 14.5 months (95% CI, 11.4-21.6) and mPFS of 7.7 months (95% CI, 6.4-9.9). Patients who had PD had an mOS of 5.5 months (95% CI, 4.4-7.9) and mPFS of 2.1 months (95% CI, 1.8-2.5). Evaluating patients with CP-A liver function, those with CR/PR, SD, and PD had an mOS of NE (95% CI, 29.7-NE), 21.6 months (95% CI, 15.6-NE), and 8.0 months (95% CI, 6.5-22.2), respectively (*P* < .0001). Among patients with CP-A, mPFS in those with CR/PR, SD, and PD was 17.7 months (95% CI, 13.3-32.4), 9.2 months (95% CI, 7.7-13.6), and 2.5 months (95% CI, 2.2-2.8), respectively (*P* < .0001). Overall and PFS by best response are shown in Supplementary Figure S1.

Mean time on treatment was longest in patients with CP-A (8.0 months), followed by CP-B (4.5 months) and CP-C (3.5 months). Adverse events (AEs) experienced by patients included fatigue in 59.9% and hypertension in 19.6%, with grade 3 or greater hypertension in 3.7%. Sixteen percent of patients experienced an immune-related AE, with 4.7% experiencing a grade 3 or greater event. Bleeding occurred in 12.1% of the patients. Of these, 15 patients had a gastrointestinal bleed that was not clearly variceal, which included 4 with grade 1/2 bleeding, 10 with grade 3/4 bleeding, and 1 patient with a grade 5 bleed (of note, 3 patients with a grade 3 gastrointestinal bleed were not on bevacizumab at the time of bleed). Seven patients experienced variceal bleeding, all of which were grade 3. None of the patients with variceal bleed underwent prior embolization and 5 patients were receiving nonselective beta blockers at the time of bleeding event. Twelve patients experienced grade 1 epistaxis, 2 patient had bleeding from a tumor (one grade 2 prior to starting therapy and the other grade 3), and 3 patients had an intracranial bleed (two grade 3 and one grade 5). Thirty-seven percent of the patients received second-line systemic therapy, with most common among these including lenvatinib (22.8%), cabozantinib (3.4%), and ipilimumab plus nivolumab (2.5%). A higher proportion of patients with CP-A received second-line therapy compared to those with CP-B liver function (41% vs 30%). No patients with CP-C liver function received second-line systemic therapy.

### Univariate and multivariate analysis for factors predictive of survival

In a univariate analysis for factors associated with OS, factors of negative prognostic value included higher CP score, higher ALBI score, non-White race, history of hepatitis B, ECOG performance status of 1 or 2, and macrovascular invasion (Table 3). Factors predictive of improved OS included prior surgery and prior embolization. In a univariate analysis for factors predictive of PFS, negative prognostic factors included CP-B8-C12, ALBI grade 2, ALBI grade 3, female sex, and ECOG performance status 2 (Supplementary Table S1). Factors predictive of improved PFS include prior embolization and prior radiotherapy.

**Table 3.** Univariate analysis for factors predictive of overall survival.

Variable	Level	HR (95% CI)	*P*-value
Child-Pugh	A5/6	Reference	<.0001
	B8-C12	6.134 (4.137, 9.094)	<.0001
	B7	2.358 (1.590, 3.497)	<.0001
ALBI Grade	Grade 1	Reference	<.0001
	Grade 3	6.109 (3.517, 10.611)	<.0001
	Grade 2	2.591 (1.814, 3.701)	<.0001
Age	Continuous	1.003 (0.988, 1.018)	.6904
Age	<65	Reference	.7325
	≥65, <75	1.149 (0.814, 1.622)	.4302
	≥75	1.081 (0.708, 1.649)	.7189
Sex	Male	Reference	
	Female	0.956 (0.656, 1.394)	.8168
Race	White	Reference	
	Non-White	1.481 (1.022, 2.146)	.0376
BMI	<25	Reference	.4519
	≥25, <30	0.937 (0.647, 1.358)	.732
	≥30	0.792 (0.545, 1.152)	.2229
Hepatitis B	No	Reference	
	Yes	1.846 (1.024, 3.329)	.0416
Hepatitis C	No	Reference	
	Yes	1.240 (0.907, 1.695)	.1778
Alcohol use	No	Reference	
	Yes	1.311 (0.942, 1.825)	.1078
MASH	No	Reference	
	Yes	0.908 (0.585, 1.411)	.6683
ECOG	0	Reference	<.0001
	2	3.665 (2.204, 6.094)	<.0001
	1	1.602 (1.145, 2.241)	.0059
Extrahepatic metastatic disease	No	Reference	
	Yes	1.090 (0.789, 1.505)	.6001
Macrovascular invasion	No	Reference	
	Yes	1.502 (1.109, 2.034)	.0085
AFP	<400	Reference	
	≥400	1.287 (0.931, 1.781)	.127
Prior surgery	No	Reference	
	Yes	0.510 (0.308, 0.842)	.0085
Prior radiotherapy	No	Reference	
	Yes	0.844 (0.538, 1.324)	.4604
Prior ablation	No	Reference	
	Yes	0.685 (0.447, 1.048)	.0809
Prior embolization	No	Reference	
	Yes	0.721 (0.528, 0.982)	.0383
Immune-related AE	No	Reference	
	Yes	1.191 (0.806, 1.761)	.3804

Abbreviations: CP, Child-Pugh; BMI, body mass index; MASH, metabolic-associated steatohepatitis; AFP, alpha fetoprotein; AE, adverse event; Tx, Treatment.

In multivariate analyses, higher CP score and ALBI grade, hepatitis B, higher ECOG PS, and macrovascular invasion were significantly associated with poorer survival. There was a significant statistical interaction between the CP score and ALBI grade (Table 4). In particular, in patients with CP-A, there was significant prognostic value of ALBI grade 1 vs grade 2. Evaluating factors predictive of PFS using multivariate analyses, factors associated with poorer PFS include higher CP score and female sex, while the only factor predictive of more favorable PFS was prior radiotherapy (Supplementary Table S2).

**Table 4.** Multivariate analysis for factors predictive of overall survival.

Variable	Comparison	HR (95% CI)		*P*-value
ALBI grade	Grade 2 vs grade 1 in CP 5-6	1.97 (1.31, 2.97)	.0138^*^	.0012
	Grade 3 vs grade 1 in CP 5-6	NE		
	Grade 3 vs grade 2 in CP 5-6	NE		
	Grade 2 vs grade 1 in CP 7	0.18 (0.04, 0.8)		.0247
	Grade 3 vs grade 1 in CP 7	0.03 (< 0.01, 0.35)		.0055
	Grade 3 vs grade 2 in CP 7	0.17 (0.02, 1.26)		.0827
	Grade 2 vs grade 1 in CP 8-12	1 451 097 (0, Inf)		.9802
	Grade 3 vs grade 1 in CP 8-12	985 122 (0, Inf)		.9807
	Grade 3 vs grade 2 in CP 8-12	0.68 (0.34, 1.35)		.2708
Hepatitis B	Yes vs no	2.83 (1.52, 5.26)	.0010	
ECOG PS	1 vs 0	1.59 (1.13, 2.24)	.0066^**^	.0085
	2 vs 0	2.16 (1.22, 3.80)		.0080
	2 vs 1	1.36 (0.79, 2.32)		.2657
Macrovascular invasion	Yes vs no	1.52 (1.11, 2.09)	.0100	

^*^Interaction *P*-value (with Child-Pugh).

^**^Overall *P*-value.

Abbreviations: CP, Child-Pugh; BMI, body mass index; NASH, nonalcoholic steatohepatitis; AFP, alpha fetoprotein; AE, adverse event; inf, infinity; PS, Performance status.

We also evaluated outcomes separately in Black/African American patients (*n* = 28). Median PFS and mOS were 6.2 and 12.8 months, respectively. Among 19 patients with CP-A liver function, mPFS and mOS were 6.2 months and 16.1 months, respectively.

## Discussion

To our knowledge, this is the largest study to report real-world outcomes of patients with advanced HCC receiving atezolizumab with bevacizumab in the first-line setting. Further, this is one of the first studies to evaluate outcomes in a Western population. While only studied in patients with CP-A liver function, atezolizumab with bevacizumab is frequently used in patients with CP-B7 liver function and beyond in clinical practice, although until this point, data regarding patient outcomes in this population have been lacking. Additionally, ALBI liver function has been more recently developed as an objective method to quantify liver function, although its use in prognostication of patients with HCC receiving atezolizumab with bevacizumab has not been widely evaluated. In this study, we were able to provide real-world data regarding patient outcomes based on these metrics.

In the present study, those with CP-A had an mOS of 21.6 months, which is comparable to the 19.2 months seen in an updated analysis from the IMbrave150 clinical trial.^10^ Patients with CP-B liver function had significantly shorter mOS at 6.4 months, which is comparable to prior small real-world studies reporting mOS of approximately 6-9 months in this group.^11-15^ In the present study, further stratification of those with CP-B liver function revealed an mOS of 9.1, 5.5, and 4.0 months in those with CP-B7, CP-B8, and CP-B9, liver function, respectively, showing a significant decline in survival benefit between those with CP-B7, and those with CP-B8 and CP-B9 liver function. In a univariate analysis, we also show CP-B7 and CP-B8-C12 to be predictive of worse survival which is consistent with prior multivariate analyses.^16^ While patients with CP-B8 and beyond may be candidates for atezolizumab plus bevacizumab, it is also important that they recognize that the survival benefit seen in IMbrave150 may be significantly reduced by baseline liver function. The same holds true for those with CP-B7 liver function, although this group still receives greater benefit than those with CP-B8 and beyond. It is important to note that we did not observe additional safety concerns in terms of AEs, immune-related AEs, or bleeding events in patients with CP-B7 liver function.

In addition to CP, we evaluated the role of ALBI liver score in assessing patient outcomes and found that those with grade 1 had the best survival of 34.9 months. Among patients with CP-A liver function, those with ALBI grade 1 had an mOS of 34.9 months compared to 14.2 months in those with ALBI grade 2, which shows that ALBI may serve a role for further stratification of prognosis in patients with CP-A liver function receiving atezolizumab with bevacizumab. When first developed, ALBI score was able to predict 2 prognostic subpopulations among patients with HCC and CP-A liver function, and we show that this holds true among patients receiving atezolizumab with bevacizumab.^7^ In multivariate analyses, we also show that ALBI grade has the ability to further predict outcomes among patients with CP-B7, which has previously been shown in a real-world analysis of patients with CP-B liver function receiving first-line atezolizumab with bevacizumab.^12^

In addition to baseline liver function, non-White race was predictive of poorer survival in univariate analysis. While the majority of patients in this study were White, which may limit statistical analysis to some extent, this is a concerning finding that merits further exploration. When looking at survival of 19 Black patients with CP-A liver function, mOS was 16.1 months, which was shorter than that observed in the overall population (21.6 months). In the 9 Black patients with CP-B liver function, mOS was 12.5 months. Prior data reporting outcomes of atezolizumab plus bevacizumab in this population has been highly limited to date. Non-White patients with HCC may experience poorer survival.^17^ The reasons for these disparities in outcomes may include genetic variations, socioeconomic status, varying access to screening, and disparities in care. One study demonstrated that Black and Hispanic patients may be more likely to be diagnosed at an advanced stage of disease, which may reflect disparities in screening.^18^ To our knowledge, this study includes the largest series of Black patients treated with atezolizumab plus bevacizumab. Future trials should investigate outcomes specifically in minority population. In our study, response to atezolizumab plus bevacizumab was highly correlated with OS. One-year survival rates for patients achieving CR/PR, SD, and PD were 86.5%, 57.6%, and 31.7%, respectively. This study suggests that response can be considered as a surrogate measure for OS in HCC with immunotherapy. Patients who have progressive disease on first-line therapy should be considered for clinical trials using novel therapeutics as they are otherwise expected to have poor outcomes.

Overall, this study demonstrates variable outcomes in patients with HCC receiving atezolizumab plus bevacizumab based on baseline liver function. A key limitation of this study rests within the intrinsic limitations of the CP score, in that 2 of its components, ascites and encephalopathy, are subjective in nature. Within our review, we attempted to determine the volume of ascites based on imaging and physical findings, and encephalopathy based on clinical documentation, which is dependent upon the assessment of the provider documenting their visit with the patient. There can be variability between providers, and these factors may also be transient. On the other hand, the ALBI score is an objective measure, which can be calculated with certainty near the time of treatment. In our study, the concordance between provider documentation and investigator-assessed CP score was only 80% suggesting high rates of miscalculation of CP score in the clinical practice. Further, the CP score was missing from the clinical notes in one-third of the patients reflecting the need for standardizing oncology notes for patients with HCC. In our study cohort, patients with CP-B and CP-C liver function who received atezolizumab with bevacizumab are likely a selected population, who were deemed fit to undergo systemic therapy despite poor liver function and may lead to overestimation of the benefit of treatment in this population. Other limitations of the study include its retrospective nature and inclusion primarily of patients at high-volume academic centers, questioning the generalizability of its conclusions. However, in this study, patients were included from the sites at 6 different states including Minnesota, Wisconsin, Iowa, Ohio, Arizona, and Florida, broadening the applicability of the study results. Patients were treated at both academic and community sites, although most of the cases were included from tertiary care centers.

## Conclusions

CP and ALBI liver function are both valuable methods to predict outcomes of patients receiving atezolizumab plus bevacizumab as first-line systemic therapy for advanced HCC. This regimen remains a viable option for patients with CP-B7 liver function with no additional safety concern, although the benefit is significantly less than those with CP-A liver function. ALBI has additional prognostic value, with 2 subpopulations with significantly different outcomes identified among patients with CP-A liver function and should be routinely calculated in clinical practice for patients receiving atezolizumab plus bevacizumab. Future trials should focus on underserved populations, especially Black patients, who may have differential outcomes.

## Supplementary material

Supplementary material is available at *The Oncologist* online.

oyae142_suppl_Supplementary_Figure

oyae142_suppl_Supplementary_Table

## Data Availability

Access to data will be made available upon request to corresponding author.
