# Supplementary material for: Atezolizumab plus bevacizumab as first-line systemic therapy for hepatocellular carcinoma: a multi-institutional cohort study
Source: Oncologist. 2024 Jul 9;29(11):986–96. doi: 10.1093/oncolo/oyae142 (PMC11546648; doi:10.1093/oncolo/oyae142)
Supplement: oyae142_suppl_Supplementary_Figure [file oyae142_suppl_supplementary_figure.pdf]

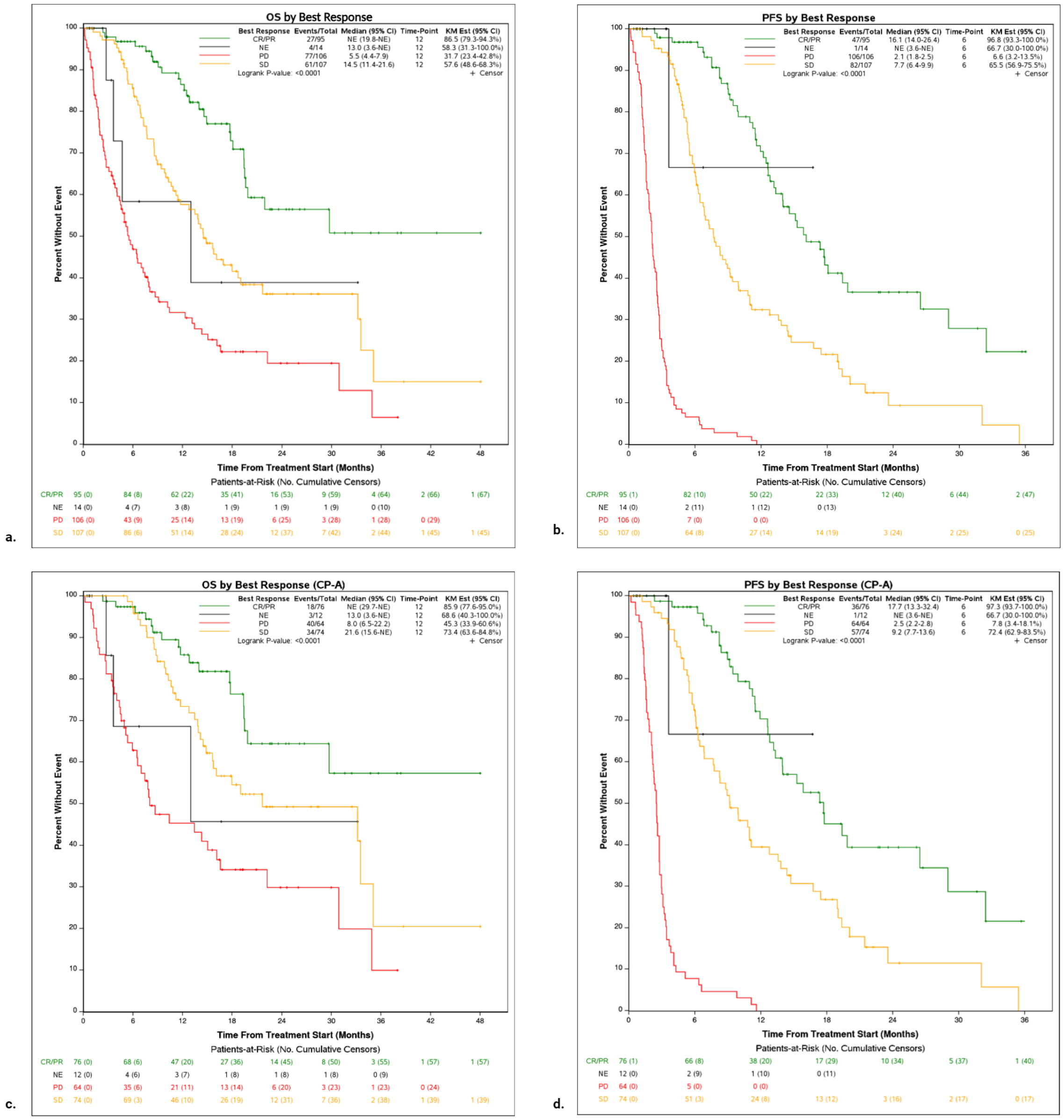

Supplemental Figure 1: a. Overall survival and **b**. progression free survival by best tumor response among all patients, and **c.** overall survival and **d.** progression free survival by best tumor response among patients with Child Pugh A liver function.

Abbreviations: CR: complete reponse, PR: Partial respons, SD: Stable disease, NE: Not evaluable
