# Supplementary material for: Atezolizumab plus bevacizumab as first-line systemic therapy for hepatocellular carcinoma: a multi-institutional cohort study
Source: Oncologist. 2024 Jul 9;29(11):986–96. doi: 10.1093/oncolo/oyae142 (PMC11546648; doi:10.1093/oncolo/oyae142)
Supplement: oyae142_suppl_Supplementary_Table [file oyae142_suppl_supplementary_table.pdf]

Supplemental Table 1: Univariate analysis for factors predictive of progression free survival

Variable	Level	HR (95% CI)	P-Value
Child Pugh	A5/6	Reference	<.0001
	B8-C12	2.939 (2.068, 4.176)	<.0001
	B7	1.259 (0.876, 1.811)	0.2136
ALBI Grade	Grade 1	Reference	0.0002
	Grade 3	2.662 (1.622, 4.370)	0.0001
	Grade 2	1.468 (1.112, 1.936)	0.0067
Age	Continuous	0.984 (0.972, 0.996)	0.0103
Age	< 65	Reference	0.3181
	≥ 65, < 75	0.843 (0.632, 1.126)	0.2488
	≥ 75	0.781 (0.549, 1.112)	0.1702
Sex	Male	Reference	
	Female	1.432 (1.060, 1.934)	0.0191
Race	Non-White	Reference	
	White	0.827 (0.593, 1.152)	0.2612
BMI	< 25	Reference	0.9038
	≥ 25, < 30	1.055 (0.770, 1.445)	0.7387
	≥ 30	0.987 (0.713, 1.367)	0.9383
Hepatitis B	No	Reference	
	Yes	1.215 (0.679, 2.174)	0.5115
Hepatitis C	No	Reference	
	Yes	1.139 (0.871, 1.490)	0.3411
EtOH	No	Reference	
	Yes	1.027 (0.770, 1.370)	0.8556
NASH	No	Reference	
	Yes	0.701 (0.472, 1.043)	0.0795
ECOG	0	Reference	0.0078
	2	1.995 (1.270, 3.136)	0.0027
	1	1.033 (0.785, 1.358)	0.8187
Extrahepatic Metastatic Disease	No	Reference	
	Yes	1.088 (0.823, 1.438)	0.5546
Macrovascular Invasion	No	Reference	
	Yes	1.160 (0.894, 1.504)	0.2648
AFP	< 400	Reference	
	≥ 400	1.097 (0.830, 1.450)	0.5166
Prior Surgery	No	Reference	
	Yes	0.953 (0.668, 1.361)	0.7926
Prior radiotherapy	No	Reference	
	Yes	0.650 (0.425, 0.994)	0.0471
Prior Ablation	No	Reference	
	Yes	0.883 (0.634, 1.230)	0.4628
Prior Embolization	No	Reference	
	Yes	0.663 (0.510, 0.863)	0.0023
Immune-related AE	No	Reference	
	Yes	0.772 (0.536, 1.112)	0.1645

Abbreviations: CP: Child Pugh, BMI: body mass index, NASH: non-alcoholic steatohepatitis, AFP: alpha fetoprotein, AE: adverse event

Supplemental Table 2: Multivariate analysis for factors predictive of progression free survival

<table>
<tr><th>Variable</th><th>Comparison</th><th>HR (95% CI)</th><th colspan="2">p-Value</th></tr>
<tr><td rowspan="3">Child Pugh</td><td>7 vs 5-6</td><td>1.36 (0.94, 1.97)</td><td rowspan="3"><0.0001*</td><td>0.0984</td></tr>
<tr><td>8-12 vs 5-6</td><td>3.14 (2.20, 4.48)</td><td><0.0001</td></tr>
<tr><td>8-12 vs 7</td><td>2.30 (1.46, 3.64)</td><td>0.0004</td></tr>
<tr><td>Sex</td><td>Female vs Male</td><td>1.60 (1.18, 2.17)</td><td colspan="2">0.0024</td></tr>
<tr><td>Prior RT</td><td>Yes vs No</td><td>0.63 (0.41, 0.97)</td><td colspan="2">0.0355</td></tr>
</table>

* Overall P-Value

Abbreviations: CP: Child Pugh, BMI: body mass index, NASH: non-alcoholic steatohepatitis, AFP: alpha fetoprotein, AE: adverse event
